# Cytokinetic diversity in mammalian cells is revealed by the characterization of endogenous anillin, Ect2 and RhoA

**DOI:** 10.1098/rsob.220247

**Published:** 2022-11-23

**Authors:** Mathieu C. Husser, Imge Ozugergin, Tiziana Resta, Vincent J. J. Martin, Alisa J. Piekny

**Affiliations:** ^1^ Biology Department, Concordia University, Montreal, Quebec, Canada; ^2^ Center for Applied Synthetic Biology, Concordia University, Montreal, Quebec, Canada; ^3^ Center for Microscopy and Cellular Imaging, Concordia University, Montreal, Quebec, Canada

**Keywords:** cytokinesis, RhoA, actomyosin, CRISPR, microscopy

## Abstract

Cytokinesis is required to physically separate the daughter cells at the end of mitosis. This crucial process requires the assembly and ingression of an actomyosin ring, which must occur with high fidelity to avoid aneuploidy and cell fate changes. Most of our knowledge of mammalian cytokinesis was generated using over-expressed transgenes in HeLa cells. Over-expression can introduce artefacts, while HeLa are cancerous human cells that have lost their epithelial identity, and the mechanisms controlling cytokinesis in these cells could be vastly different from other cell types. Here, we tagged endogenous anillin, Ect2 and RhoA with mNeonGreen and characterized their localization during cytokinesis for the first time in live human cells. Comparing anillin localization in multiple cell types revealed cytokinetic diversity with differences in the duration and symmetry of ring closure, and the timing of cortical recruitment. Our findings show that the breadth of anillin correlates with the rate of ring closure, and support models where cell size or ploidy affects the cortical organization, and intrinsic mechanisms control the symmetry of ring closure. This work highlights the need to study cytokinesis in more diverse cell types, which will be facilitated by the reagents generated for this study.

## Graphical abstract

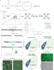

## 1. Introduction

**Figure 1.**
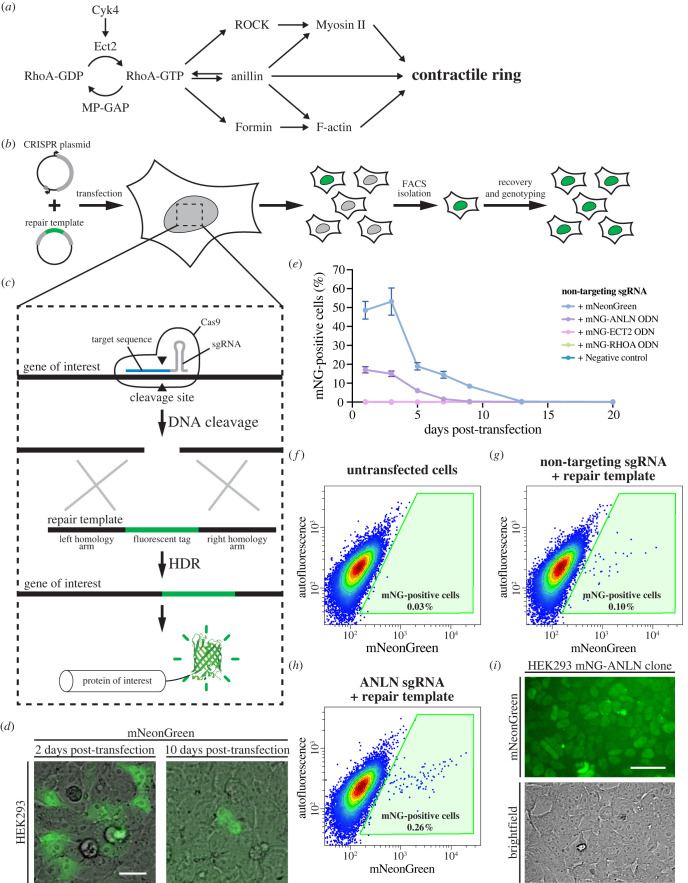
Endogenous tagging of cytokinesis proteins in human cells using CRISPR/Cas9. (*a*) Diagram showing the core pathway regulating contractile ring assembly during cytokinesis. The generation of active RhoA (RhoA-GTP) by the GEF Ect2 at the equatorial cortex is required for ring assembly. Active RhoA recruits and/or activates effectors (anillin, formin and ROCK proteins) to assemble actomyosin filaments into the contractile ring. (*b*) The experimental workflow used for endogenous tagging is shown. Cultured cells were transfected with a CRISPR plasmid and a repair template to express the CRISPR/Cas9 components and integrate the coding sequence encoding a fluorescent protein. Some cells started to express the fluorescent protein after several days and were isolated by FACS. Clonal cell lines were then recovered and genotyped to verify the edits. (*c*) The mechanism of endogenous tagging by CRISPR/Cas9 is shown. A gene-specific sgRNA directs DNA cleavage by Cas9 at the target site. The DSB generated by Cas9 can be repaired by HDR using the provided repair template and integrate the coding sequence for a fluorescent protein at the target site. This will result in the expression of the target protein fused to a fluorescent protein. (*d*) Representative images show ectopic mNeonGreen expression 2 days after transfection (left) and nuclear mNeonGreen-anillin signal 10 days after transfection (right). The scale bar is 25 µm. (*e*) The graph shows the percentage of mNeonGreen-positive cells over time after transfection with different mNeonGreen repair templates as indicated, assessed by flow cytometry. A constitutive mNeonGreen expression vector was used as a positive control compared with the mNeonGreen-anillin, mNeonGreen-Ect2 and mNeonGreen-RhoA repair templates, and an empty plasmid as a negative control. (*f–h*) Representative flow cytometry plots show mNeonGreen fluorescence in HEK293 cells 13 days after transfection with constructs designed to tag anillin with mNeonGreen. Non-transfected cells (*f*) were used to remove non-fluorescent cells. A negative control lacking a sgRNA (*g*) shows residual ectopic expression of mNeonGreen from the transfection of a repair template alone, while (*h*) shows cells transfected with both the ANLN-targeting CRISPR plasmid and repair template. The gate shown in green was used to isolate tagged cells by FACS. (*i*) A representative image shows a colony of mNeonGreen-anillin tagged HEK293 cells after single-cell isolation and recovery. The scale bar is 50 µm.

Cytokinesis describes the physical separation of a cell into two daughters, which occurs at the end of mitosis. This process must be tightly spatio-temporally controlled as failure can cause changes in cell fate and disease [1,2]. Cytokinesis occurs via the assembly and ingression of a RhoA-dependent contractile ring that constricts to pull in the overlying plasma membrane. Multiple pathways regulate ring assembly in cultured cells and model organisms [3–6]. These pathways ensure that active RhoA is enriched in the equatorial plane to assemble the ring. Ect2 is the guanine nucleotide exchange factor (GEF) that activates RhoA during cytokinesis and requires binding to phospholipids and the central spindle protein Cyk4 (MgcRacGAP) for its activity (figure 1*a*; [7–9]). The depletion of Cyk4 or Ect2 in HeLa cells prevents the accumulation of active RhoA at the equatorial cortex and leads to cytokinesis failure [9–13]. Active RhoA (RhoA-GTP) recruits and activates effectors, including formin and RhoA kinase (ROCK), to generate actomyosin filaments and assemble the ring (figure 1*a*; [6,14]). Anillin is also recruited by active RhoA and acts as a scaffold protein that tethers the ring to the plasma membrane [3,15–17]. In support of its cross-linking function, the depletion of anillin in HeLa or S2 cells leads to ring oscillations and cytokinesis failure [15,16,18]. Anillin may also be involved in the retention of active RhoA at the equatorial cortex, as well as its removal during constriction [19–21]. Multiple mechanisms control this core cytokinesis machinery in different model systems, yet their relative requirement remains unknown [4].

Multiple pathways regulate cytokinesis. The central spindle facilitates the recruitment and activation of Ect2 in proximity to the equatorial cortex where it generates active RhoA [8,9,22–31]. Astral microtubules also define the cleavage plane by removing cortical regulators from the poles [32–37], while spindle-independent pathways polarize the cortex through signals associated with chromatin, centrosomes and kinetochores [5,37–46]. For example, studies showed that chromatin-associated active Ran (Ran-GTP; Ras-related nuclear protein) coordinates the position of the ring with segregating chromosomes [5,38,39,43,44]. These pathways also act in concert with the negative regulator of RhoA, MP-GAP (M-phase GTPase-activating protein), which is globally localized [37]. Having multiple mechanisms to control the function of cortical regulators ensures robust cytokinesis, but their requirement is expected to differ with cell type.

Studies in different organisms and tissues have revealed differences in the regulators of cytokinesis [33,34,36,40, 44,45,47–49]. For example, anillin is essential for cytokinesis in cultured cells (HeLa and *Drosophila* S2 cells), but not in the early *Caenorhabditis elegans* zygote, and Dalmatian dogs carrying an anillin truncation mutant did not have obvious cell division defects [15,18,50,51]. However, later in *C. elegans* development, neuronal precursor cells require anillin for cytokinesis [48,52]. Thus, the mechanisms regulating cytokinesis vary with cell fate, but also with other parameters including size, shape and ploidy. In the two-cell *C. elegans* embryo, the somatic AB and germline P_1_ cells have different ring assembly and ingression kinetics with different levels of myosin in the ring [44]. In the four-cell *C. elegans* embryo, the ABa and ABp cells have stronger requirements for formin-derived F-actin compared to EMS or P_2_ cells, which are regulated by cell-extrinsic and intrinsic factors, respectively [47]. The observed cytokinetic diversity in different cell types highlights the importance of understanding how mechanisms regulating cytokinesis vary during development. However, most of our knowledge of human cell cytokinesis is derived from the over-expression of transgenes in HeLa cells, which are cancerous in origin and have lost their epithelial identity. Given the vast number of human cells with specialized functions, it is likely that the mechanisms regulating cytokinesis will vary with cell type. For example, liver hepatocytes abort cytokinesis to gain ploidy for their function [2,53,54], while the ring closes asymmetrically in epithelial cells, which could help them retain apicobasal polarity and be properly positioned after division [55–58]. Understanding how these different modes of cytokinesis are controlled can help us understand and/or treat cytokinesis-related pathologies.

Gene editing tools provide an opportunity to study proteins in diverse cell types [4,59]. In human cells, cytokinesis has mostly been studied using over-expressed transgenes fused to fluorescent tags for visualization and/or affinity tags for biochemical assays. In HeLa cells, the localization of endogenous anillin fixed and stained with antibodies is similar to anillin over-expression. However, Ect2 and RhoA show inconsistent localization patterns and/or cause cytokinesis phenotypes when over-expressed [9,15,60]. TCA-fixation-based immunofluorescence microscopy is still one of the most reliable methods to visualize the enrichment of RhoA at the equatorial cortex [9,61–63], and Ect2 over-expression can lead to cytokinesis failure [60]. In addition, measurements can be confounded by the variability in transgene expression between transfected cells, whereas endogenous probes enable more quantitative measurements. There are also several examples where endogenously tagged proteins cause fewer phenotypes compared to proteins from over-expressed transgenes, which may not fold properly, fail to assemble into complexes and/or disrupt downstream processes [4,64–66]. Moreover, the same tools can be used to introduce genetic edits into different cell lines derived from the same organism.

The most widely used tool for gene editing is CRISPR/Cas9 (clustered regularly interspaced short palindromic repeats/CRISPR-associated protein 9) comprised of Cas9 nuclease and a sgRNA (single guide RNA), which contains a 20-nucleotide target sequence that corresponds to a genomic target site (figure 1*b*,*c*; [67–70]). Cas9 is targeted to this site by the sgRNA and cleaves the DNA to introduce a double-stranded break (DSB). Human cells typically repair DSBs by non-homologous end joining (NHEJ), but can also use the homology-directed repair (HDR) pathway, which makes use of a homologous repair template to fill in the gap where the DSB was introduced [71,72]. To introduce a fluorescent marker at a precise location in the genome of human cells, CRISPR/Cas9 can be used along with a synthetic repair template designed to carry the fluorescent marker flanked with homology arms for HDR (figure 1*c*; [73]). When introduced in frame with a gene, the fluorescent marker will be expressed as a fusion with the protein of interest. Efforts to share validated tools for endogenous tagging (sgRNA sequence and repair template) have made these tags more readily accessible and easy to use ([74–79]; Allencell.org; Addgene.org). However, despite these shared resources and the advantages to using endogenous tags, few cytokinesis proteins have been tagged endogenously in human cells [66,80,81].

In this work, we generated reagents to endogenously tag anillin, Ect2 and RhoA with mNeonGreen using CRISPR/Cas9 gene editing, and re-purposed existing constructs to tag cellular markers with different fluorescent proteins. These reagents were used to characterize the spatio-temporal localization of endogenously tagged anillin, Ect2 and RhoA during cytokinesis in HeLa cells for the first time. We then tagged endogenous anillin with mNeonGreen in multiple mammalian cell lines, including HCT116, HepG2 and MDCK, which are difficult to genetically modify and have not been used for cytokinesis studies before. In-depth localization studies of anillin revealed cytokinetic diversity among the cell types. Specifically, we found differences in the breadth, enrichment and timing of the cortical localization of anillin. By making these tools available to the cell biology community, we hope to fuel new studies of the mechanisms regulating cytokinesis in diverse cell types.

## 2. Results

### 2.1. Endogenous tagging of cytokinesis proteins and cellular markers

To study cytokinesis regulators in their native cellular environment, we generated reagents to endogenously tag anillin (ANLN), Ect2 (ECT2) and RhoA (RHOA) with mNeonGreen in human cells using CRISPR/Cas9 (figure 1*a–c*). We designed sgRNAs to target the ANLN, ECT2 and RHOA genes, and corresponding repair templates to integrate the coding sequence of mNeonGreen in frame with their coding sequences. The repair templates were designed to have 1 kb homology arms flanking the mNeonGreen gene. Three sgRNAs were designed for each gene (electronic supplementary material, table S1) and cloned into a plasmid expressing the high-specificity HypaCas9 protein. We only targeted the N-terminus of RhoA because the C-terminus is post-translationally cleaved, but we targeted both ends of anillin and Ect2 for tagging. The sets of sgRNA-containing CRISPR plasmids and repair templates were validated by co-transfecting them into HEK293 cells (immortalized human female embryonic kidney cells with 64 modal chromosomes; hypotriploid) and monitoring the appearance of a mNeonGreen signal with the expected localization patterns.

When generating endogenously tagged cell lines, we observed non-specific ectopic expression of mNeonGreen directly following transfection (figure 1*d*). This ectopic signal has been reported previously [82], and likely arises from ectopic expression from the repair template rather than genomic integration, as it progressively disappears over approximately 9 days following transfection (figure 1*e*). We observed the expected localization pattern for the tagged proteins starting approximately 4 days after transfection.

Using the workflow in figure 1*b*, we generated endogenous N-terminal mNeonGreen fusions of anillin, Ect2 and RhoA in HEK293 cells. Notably, we did not recover cells with C-terminal tags fused to anillin, while cells with C-terminal tags fused to Ect2 did not show the correct cellular localization (data not shown). The C-terminal tag likely disrupts the function of Ect2, but it is not clear why the tagging was unsuccessful for the C-terminal end of anillin. The successful repair templates and optimal sgRNAs used for each locus are listed in table 1. After confirming each tag by visual inspection, single fluorescent cells were isolated by FACS (fluorescence-activated cell sorting; e.g. figure 1*f–h*). Single-cell clones were screened by fluorescence microscopy (e.g. figure 1*i*), PCR and sequencing (e.g. electronic supplementary material, figure S1) to validate the cell lines. Finally, this workflow was repeated to generate HeLa (human female cervical carcinoma with 82 modal chromosomes; hypertriploid) cell lines expressing mNeonGreen-tagged anillin, Ect2 and RhoA. We were able to isolate HEK293 and HeLa cell lines where tagged anillin and Ect2 were homozygous. However, we were unable to isolate homozygous tagged RhoA cell lines despite screening 72 clones of RhoA-tagged HEK293 and 24 clones of RhoA-tagged HeLa. This may be due to the low tagging efficiency at this locus, or some impediment to RhoA function caused by the tag.

**Table 1.** A toolkit of repair templates to tag multiple proteins and cellular components. The repair templates generated and/or used in this study are listed. For each protein or cellular component, the terminus targeted for tagging, the protein linker sequence and the available fluorescent proteins are indicated. The best sgRNA sequence used for CRISPR targeting of each locus is also indicated. The cell lines that each repair template has been tested in and the Addgene ID for each repair template are also listed. Asterisks indicate repair templates that were created and tested in WTC-11 iPSCs by the Allen Institute for Cell Science (obtained from Addgene [76]; Allencell.org).

protein/structure	gene/locus	fluorescent protein	terminus	protein linker	sgRNA sequence/PAM	cell lines tested	addgene ID (repair template)	addgene ID (CRISPR plasmid)
Anillin	ANLN	mNeonGreen	N-	GGSGGS	GTCTCGTAGTCCGACGCCTG/GGG	HEK293, HeLa, HCT116, HepG2	183 834	183 874/183 873
Ect2	ECT2	mNeonGreen	N-	GGSGGS	TATTAACATCCACTACTGGG/AGG	HEK293	183 835	183 876/183 875
RhoA	RHOA	mNeonGreen	N-	GGSGGS	AATCACCAGTTTCTTCCGGA/TGG	HEK293	183 836	183 878/183 877
anillin	CfANLN	mNeonGreen	N-	GGSGGS	GGCGATGGACCCGTTTACCG/AGG	MDCK	183 837	183 880/183 879
H2B histone	H2BC11	mEGFP	C-	DPPVAT	ACTCACTGTTTACTTAGCGC/TGG	WTC-11*, HEK293	10 9121*	183 883/183 881
		mRuby2				HEK293	18 3866	
		TagBFP				HEK293	18 3867	
actin beta	ACTB	mEGFP	N-	AGSGT	GCCGTTGTCGACGACGAGCG/CGG	WTC-11*, HEK293	87 425*	183 885/183 884
		mRuby2				HEK293	18 3868	
myosin IIB	MYH10	mEGFP	N-	YSDLELKLRIP	GTTCTCTGCGCCATTGTAAA/TGG	WTC-11*	87 428*	183 887/183 886
		mRuby2				—	18 3869	
tubulin alpha	TUBA1B	TagRFP-T	N-	GGSGGS	GATGCACTCACGCTGCGGGA/AGG	WTC-11*, HEK293	10 1785*	183 889/183 888
plasma membrane	AAVS1	mNeonGreen-CAAX	—	—	GGGGCCACTAGGGACAGGAT/TGG	HEK293	18 3870	183 891/183 890
		mRuby2-CAAX				HEK293	18 3871	

To expand on this toolset, we obtained repair templates and sgRNA sequences from the Allen Institute for Cell Science (Addgene) and replaced the mEGFP tag with either a red (mRuby2) or a blue (TagBFP) fluorophore. We also generated repair templates targeted to the AAVS1 locus (Adeno-Associated Virus Integration Site 1) to express membrane-specific tags by fusing mNeonGreen or mRuby2 with the CAAX domain of K-Ras [83]. The repair templates and sgRNAs that were generated and used in this study are listed in table 1. Notably, we were unable to obtain MYH10-tagged cells after several attempts using four different sgRNAs in HEK293 and HeLa cells. Since this protein was tagged successfully in iPSCs [76], there could be cell-specific differences in how this gene is expressed, or in the efficiency of repair at this locus. However, the other constructs were used to successfully generate multiple HEK293 cell lines expressing different combinations of endogenous tags (electronic supplementary material, figure S2). We generated double-tagged H2B-TagBFP; mNeonGreen-anillin cells, which were then used to generate triple-tagged lines by also tagging the plasma membrane or β-actin with mRuby2 (electronic supplementary material, figure S2A,B). We found no major differences in the growth and viability of the edited lines over 3–4 generation times (5 days) compared to non-edited HEK293 cells (electronic supplementary material, figure S2C). We also found that the furrow peaks were well-aligned between double-tagged and single-tagged cells (electronic supplementary material, figure S2D), showing that these cells can be used for localization studies when multiple components are tagged.

### 2.2. Endogenous tags are preferable for localization studies compared to transgenes

**Figure 2.**
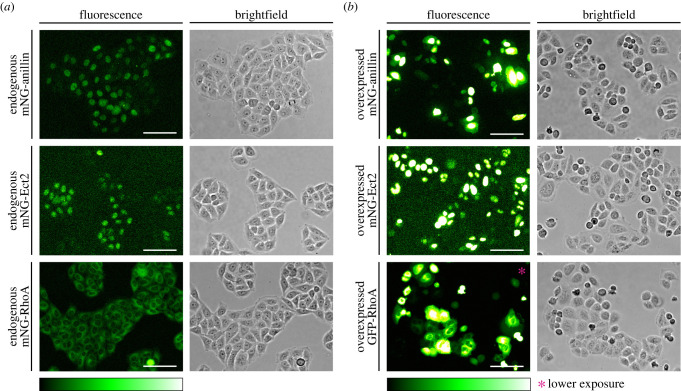
Endogenous tags are more reliable than transient over-expression. (*a*) Fluorescent (left) and corresponding brightfield (right) images of HeLa cells show anillin (top), Ect2 (middle) and RhoA (bottom) endogenously tagged with mNeonGreen. (*b*) Fluorescent (left) and corresponding brightfield (right) images of HeLa cells show exogenous expression of mNeonGreen-anillin (top), mNeonGreen-Ect2 (middle) and GFP-RhoA (bottom) 24 h after transfection. *Image taken with a lower exposure time. The scale bars are 100 µm. The relative intensity of mNeonGreen is shown in the corresponding scale.

We then characterized the localization of endogenous mNeonGreen-tagged anillin, Ect2 and RhoA in live HeLa cells by fluorescence microscopy for the first time. To emphasize the drastic differences in expression between endogenous tags and transiently over-expressed transgenes, we compared the levels of fluorescent proteins in multiple cells within fields of view. Indeed, there was a striking difference in the levels and variability across cell populations where fluorescent protein tags were transiently over-expressed compared to endogenous tags, which were weaker and more uniform (figure 2*a*,*b*). While there was some variability in anillin and Ect2 expression levels in the endogenously tagged cell lines, this was expected due to their cell cycle-dependent turnover, and was much lower compared to cells where they were over-expressed (figure 2*a*,*b*). The transfected cells also showed variable morphology with more rounded cells, likely due to apoptosis, and/or cell cycle phenotypes (figure 2*b*). We also observed binucleate cells indicative of cytokinesis failure with Ect2 and RhoA over-expression (figure 2*b*). These data show that the endogenous tagging of cytokinesis proteins provides more accurate readouts of protein expression compared to over-expressed transgenes, which can also cause cytokinesis phenotypes. This comparison reveals the importance of considering expression levels when generating stable cell lines from transgenes, as the lines chosen for further study could be extremely different compared to the endogenous proteins.

### 2.3. Core cytokinesis regulators have distinct spatio-temporal distributions

**Figure 3.**
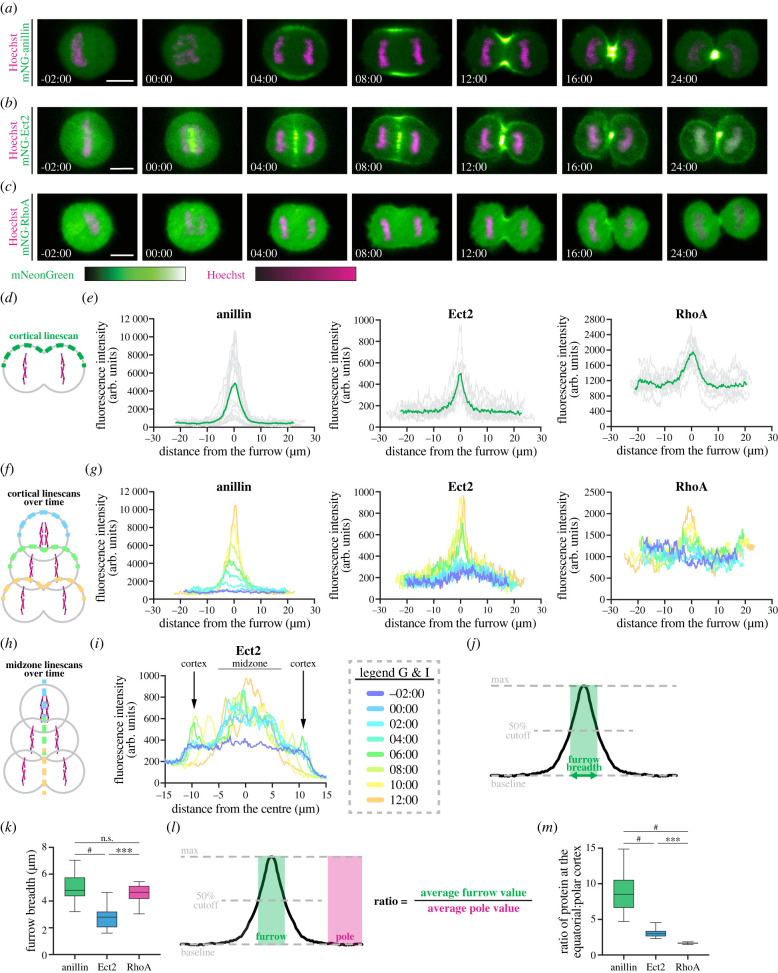
Endogenous anillin, Ect2 and RhoA show distinct localization patterns during cytokinesis in HeLa cells. (*a–c*) Time-lapse images show cells expressing endogenous mNeonGreen-anillin (*a*), mNeonGreen-Ect2 (*b*) and mNeonGreen-RhoA (*c*) during cytokinesis. mNeonGreen is shown in green and DNA (stained with Hoechst) in magenta. Times are shown in minutes and seconds relative to anaphase onset. The scale bar is 10 µm. The relative intensities of mNeonGreen and Hoechst are shown in the corresponding scales. (*d–e*) A schematic (*d*) shows the location of the cortical linescan used to plot the intensity of fluorescence at the onset of furrowing ((*e*), 8–12 min after anaphase onset) in HeLa cells expressing endogenously tagged anillin (left, *n* = 16), Ect2 (middle, *n* = 10) and RhoA (right, *n* = 10). Individual replicates are shown in grey, and the average is shown in green. (*f–g*) A schematic (*f*) shows the location and timing of the linescans used to plot the fluorescence intensity along the cortex in single HeLa cells (*g*) expressing endogenously tagged anillin (left), Ect2 (middle) and RhoA (right) at multiple timepoints starting 2 min before anaphase onset, and shown in different colours as indicated in the scale below. (*h–i*) A schematic (*h*) shows the location and timing of the linescans used to plot the fluorescent intensity along the midzone of a single HeLa cell (*i*) expressing endogenously tagged Ect2 at multiple timepoints starting 2 min before anaphase onset, shown in the same colours as *g*. (*j*) A schematic shows how the breadth at the equatorial cortex was calculated for *k*. (*k*) Box plots show the breadth of anillin (*n* = 16), Ect2 (*n* = 10) and RhoA (*n* = 10) in HeLa cells. (*l*) A schematic shows how the ratio of protein at the equatorial cortex (furrow) relative to the polar cortex was calculated for (*m*). (*m*) Box plots show the enrichment of anillin (*n* = 16), Ect2 (*n* = 10) and RhoA (*n* = 10) at the equatorial cortex in HeLa cells. Box plots in (*k*) and (*m*) show the median line, quartile box edges and minimum and maximum value whiskers. Statistical significance was determined by one-way ANOVA (n.s., not significant; * *p* ≤ 0.05; ** *p* ≤ 0.01; *** *p* ≤ 0.001; ^#^ *p* ≤ 0.0001).

We then imaged cells during cytokinesis to follow the localization of endogenously tagged anillin, Ect2 and RhoA (figure 3*a*–*c*). As expected, anillin was enriched at the equatorial cortex shortly after anaphase onset and remained in the furrow throughout ingression, after which it localized to the midbody (figure 3*a*). We measured this enrichment using a linescan to plot the intensity of mNeonGreen-anillin along the cortex of cells at the start of furrowing (approx. 9–14 min after anaphase onset; figure 3*d*,*e*, left). To visualize localization over time, we repeated this analysis every 2 min, starting 2 min before anaphase onset and until furrowing appeared to be completed (figure 3*f*,*g*, left). We observed an increase in the enrichment and a gradual decrease in the breadth of anillin at the equatorial cortex over time, with the enrichment being first visible 4–6 min after anaphase onset (figure 3*g*, left). Ect2 initially localized to the central spindle, then was also visible at the equatorial cortex and remained in both locations during ingression, followed by its localization to the midbody (figure 3*b*; electronic supplementary material, figure S3A). We used linescans to measure the intensity of mNeonGreen-Ect2 along the cortex at furrow initiation (figure 3*e*, middle), and along the cortex and central spindle over time (figure 3*g*, middle, *h*,*i*). The localization of Ect2 to the central spindle preceded the equatorial cortex, which was first visible approximately 6 min after anaphase onset and was narrow compared to anillin. Lastly, we found that RhoA was also enriched at the equatorial cortex at the onset of ingression (figure 3*c*,*e*, right). Linescans revealed that this enrichment was visible approximately 6–8 min after anaphase onset (figure 3*c*,*g*, right). This enrichment appeared weak compared to Ect2 and anillin, likely because of the large pool of cytoplasmic RhoA. To demonstrate that mNeonGreen-RhoA can be activated, we transiently over-expressed the C-terminus of Ect2 (Ect2 (C-term); amino acids 420–882), which contains the DH domain required for nucleotide exchange and activation of RhoA [9]. In interphase and metaphase cells expressing mScarlet-I-Ect2 (C-term), we observed an increase in the cortical localization of mNeonGreen-RhoA, which reflects an increase in activity since the active form is membrane-localized (electronic supplementary material, figure S3B,C [9]).

Next, to compare the localization of anillin, Ect2 and RhoA during cytokinesis, we quantified their breadth and accumulation. To measure breadth, we used the linescans to determine the number of pixels above 50% of the normalized peak intensity (figure 3*j*) and found that while the breadth of anillin and RhoA was similar (4.9 ± 1.1 and 4.5 ± 0.8 µm, respectively), Ect2 was narrower (2.8 ± 0.9 µm; figure 3*k*). Similar results were obtained when measuring breadth as a ratio of cortical length to control for variations in cell size (electronic supplementary material, figure S4A). To measure accumulation, the ratio of the average pixel intensity of anillin, Ect2 and RhoA in the furrow relative to the polar cortex was determined (figure 3*l*). Our data revealed that anillin was most enriched, with 8.9 ± 2.9-fold (*n* = 16) more protein in the furrow than at the poles compared to Ect2 (3.1 ± 0.7-fold, *n* = 10) and RhoA (1.7 ± 0.1-fold, *n* = 10; figure 3*m*).

### 2.4. Endogenous tagging of anillin in different mammalian cell lines

Next, we tagged anillin with mNeonGreen in different cell lines to facilitate cytokinesis studies. In addition to the tagged HEK293 and HeLa cells described earlier, we tagged anillin with mNeonGreen in HCT116 (human male colon cancer with 45 modal chromosomes; near diploid), HepG2 (human male hepatoblastoma with 55 modal chromosomes, hyperdiploid) and MDCK (Madin–Darby canine female kidney with 78 or 87–90 modal chromosomes; diploid—hyperdiploid) cells. Since MDCK cells are not human, we created new sgRNAs and repair templates suited for the *Canis lupus familiaris* genome (table 1). These cell lines are rarely used for gene editing and presented distinct challenges. HCT116 and MDCK cells have low transfection efficiency using liposome-based methods, requiring nucleofection to increase transfection efficiency and recover successfully tagged cells. We also used NHEJ inhibitors to increase the efficiency of integration by HDR. Another limitation was the rate of single-cell recovery after FACS. While HeLa and MDCK cells recovered well from single-cell isolation (greater than 90% recovery, data not shown), HepG2 cells showed very low recovery and we initially only recovered two non-fluorescent clones from 120 isolated HepG2 cells. To resolve this issue, we enriched tagged cells by sorting 15 000 fluorescent cells together as a population and allowing them to recover for a few days. Then, we isolated clones by sorting 10–25 fluorescent cells per well of a 96-well plate and monitoring for the recovery of a single colony in each well. Altogether, these methods can greatly facilitate gene editing in cell lines that are difficult to edit. By incorporating these methods into our editing protocols, we successfully obtained multiple heterozygous and homozygous HeLa, HEK293, HCT116, HepG2 and MDCK cell lines where anillin was endogenously tagged with mNeonGreen.

### 2.5. Differences in anillin localization correlate with distinct ring closure kinetics

**Figure 4.**
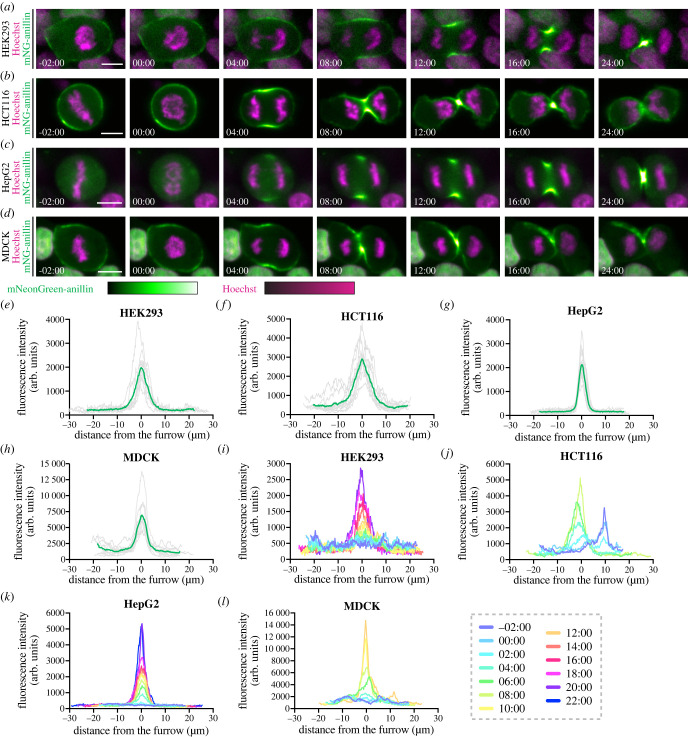
Endogenous tagging of anillin in different cell lines reveals differences in its localization during cytokinesis. (*a–d*) Time-lapse images show endogenous mNeonGreen-anillin in HEK293 (*a*), HCT116 (*b*), HepG2 (*c*) and MDCK (*d*) cells during cytokinesis. mNeonGreen is shown in green and DNA (stained with Hoechst) is in magenta. Times are shown in minutes and seconds relative to anaphase onset. The scale bar is 10 µm. The relative intensities of mNeonGreen-anillin and Hoechst are shown in the corresponding scales. (*e–h*) Graphs show fluorescence intensity of mNeonGreen-anillin along the cortex of HEK293 ((*e*), *n* = 9), HCT116 ((*f*), *n* = 11), HepG2 ((*g*), *n* = 13) and MDCK ((*h*), *n* = 10) cells at furrow initiation. (*i–l*) Graphs show fluorescence intensity of mNeonGreen-anillin along the cortex of a single HEK293 (*i*), HCT116 (*j*), HepG2 (*k*) and MDCK (*l*) cell at multiple timepoints starting 2 min before anaphase onset, shown in different colours as indicated by the scale.

To reveal cytokinetic diversity, we compared differences in anillin localization between the newly generated mNeonGreen-anillin tagged cell lines. We observed differences in when anillin was first visible at the cortex, the breadth of anillin at the equatorial cortex and the duration of ingression after anaphase onset. For example, mNeonGreen-anillin localized to the cortex in HEK293, HCT116 and MDCK cells during metaphase, but not in HeLa and HepG2 cells, where it was strictly cytosolic (figures 3*a* and 4*a–d*; −02 : 00 timepoint). All cell lines showed mNeonGreen-anillin enrichment in the equatorial cortex approximately 4–8 min after anaphase onset, where it remained in the furrow throughout ingression (figures 3*a* and 4*a–d*). However, linescans of mNeonGreen-anillin along the cortex at furrow initiation (figures 3*e*, left and 4*e–h*), and every 2 min from just before anaphase until the end of furrowing, revealed differences in the breadth of anillin between the cell lines (figures 3*g*, left and 4*i–l*). While anillin peaks were broad in HEK293, HCT116 and MDCK cells, anillin was narrow in HepG2 cells (figure 4). Also, furrowing appeared to take much longer in HepG2 and HEK293 cells compared to the other cell lines (figures 3*a*,*g* and 4*a–d*,*i–l*).

**Figure 5.**
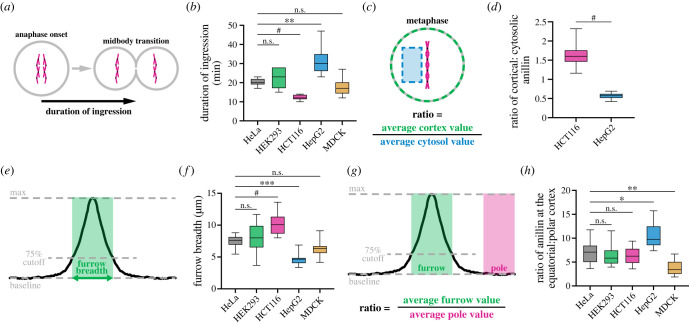
Breadth of anillin but not cumulative levels correlate with the efficiency of ring closure. (*a*) A schematic shows how the duration of ingression was measured in (*b*). (*b*) A box plot shows the duration of ingression in HeLa (*n* = 11), HEK293 (*n* = 7), HCT116 (*n* = 8), HepG2 (*n* = 11) and MDCK (*n* = 6) cells. (*c*) A schematic shows how the ratio of cortical to cytosolic mNeonGreen-anillin was measured for (*d*). (*d*) A box plot shows the enrichment of mNeonGreen-anillin at the cortex relative to the cytosol during metaphase in HCT116 (*n* = 8) and HepG2 (*n* = 12) cells. (*e*) A schematic shows how the breadth at the equatorial cortex was calculated for (*f*). (*f*) A box plot shows the breadth of mNeonGreen-anillin localization along the equatorial cortex at furrow initiation in HeLa (*n* = 16), HEK293 (*n* = 9), HCT116 (*n* = 11), HepG2 (*n* = 13) and MDCK (*n* = 10) cells. (*g*) A schematic shows how the ratio of protein in the furrow relative to the polar cortex was calculated for (*h*). (*h*) A box plot shows the ratio of anillin at the equatorial cortex compared to the polar cortex in HeLa (*n* = 16), HEK293 (*n* = 9), HCT116 (*n* = 11), HepG2 (*n* = 13) and MDCK (*n* = 10) cells. Box plots in (*b*), (*d*), (*f*) and (*h*) show the median line, quartile box edges and minimum and maximum value whiskers. Statistical significance was determined by one-way ANOVA in (*b*), (*f*) and (*h*), and Welch *t* test in (*d*) (n.s., not significant; * *p* ≤ 0.05; ** *p* ≤ 0.01; *** *p* ≤ 0.001; ^#^ *p* ≤ 0.0001).

Next, we quantified the differences in anillin localization and ring closure, using furrow ingression as a proxy, among the cell lines. First, we compared the duration of ring closure between the cell lines, starting from anaphase onset until the membrane appeared to be fully closed (figure 5*a*,*b*). While ingression took similar amounts of time on average in HeLa (20.4 ± 1.7 min, *n* = 11), HEK293 (22.3 ± 5.2 min, *n* = 7) and MDCK cells (17.7 ± 5.0 min, *n* = 6; figure 5*b*), HepG2 cells ingressed slower (31.0 ± 7.0 min, *n* = 11) and HCT116 cells ingressed faster (12.3 ± 1.4 min, *n* = 8). We also noted more variability in the duration of ingression in HEK293 and HepG2 cells compared to the other cell types.

Next, we quantified the cortical localization of anillin prior to anaphase between HCT116 and HepG2 cells, where the difference was most striking. To do this, we measured the ratio of cortical to cytosolic mNeonGreen-anillin during metaphase (figure 5*c*,*d*). Indeed, anillin was significantly enriched at the cortex during metaphase in HCT116 cells (1.6 ± 0.3-fold), but not in HepG2 cells (0.6 ± 0.1-fold; figure 5*d*). Then, we compared the breadth of anillin localization at the onset of furrowing in the different cell lines (figure 5*e*,*f*). Since anillin localized to the furrow as a well-defined peak across all cell lines, we measured breadth as the number of pixels above 75% of the normalized peak intensity (figure 5*e*,*f*). The mNeonGreen-anillin furrow was the broadest in HCT116 cells, narrowest in HepG2 cells, and was similar between HeLa, HEK293 and MDCK cells (figure 5*f*). Similar results were obtained when measuring breadth as a percentage of cortical length, showing that these differences were independent of cell size (electronic supplementary material, figure S4B). mNeonGreen-anillin was similarly enriched in the equatorial cortex of HeLa, HEK293 and HCT116 cells (7.1 ± 2.4, 6.3 ± 2.3 and 6.3 ± 1.9-fold enrichment, respectively), while HepG2 cells had a significantly stronger enrichment (10.4 ± 2.6-fold), and MDCK cells had a weaker enrichment (3.8 ± 1.5-fold; figure 5*h*). These data suggest that the breadth of anillin localization rather than accumulated levels correlate with the rate of ingression. This is further supported by measurements of two distinct populations of cells within the mNeonGreen-anillin HeLa cell line, which display higher or lower expression levels (electronic supplementary material, figure S5). Since our genotyping revealed no untagged alleles, we presume that this difference in anillin levels is due to aneuploidy causing heterogeneity and changes in gene expression. Comparing the same parameters in these two populations of cells revealed no difference in the duration of ingression and breadth of anillin (electronic supplementary material, figure S5B–F), but the accumulated levels were higher in one population compared to the other (electronic supplementary material, figure S5G,H).

### 2.6. Asymmetric ingression in MDCK cells is intrinsically controlled

**Figure 6.**
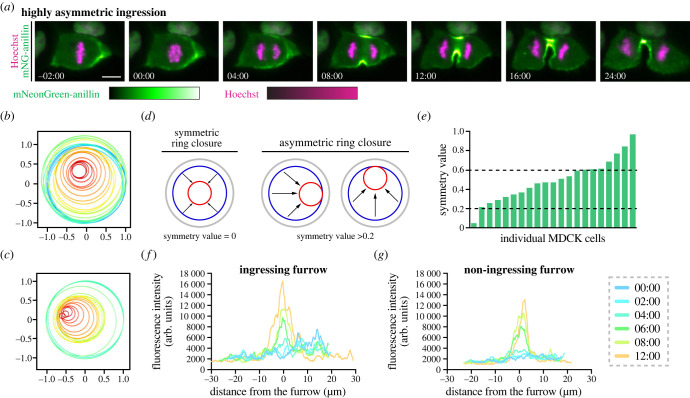
MDCK cells ingress asymmetrically. (*a*) Time-lapse images show endogenous mNeonGreen-anillin localization in different MDCK cells during cytokinesis where ingression appeared to be highly asymmetric. mNeonGreen is shown in green and DNA (stained by Hoechst) is in magenta. The relative intensities of mNeonGreen and Hoechst are shown in the corresponding scales. Times are shown in minutes and seconds from anaphase onset. The scale bars are 10 µm. (*b*,*c*) Graphs shows the position of the ring over time in cells that undergoes asymmetric (*b*) or highly asymmetric (*c*) ring closure. (*d*) Schematics show the relative position of the ring as it closes symmetrically (left; values less than 0.2) or asymmetrically (right; values greater than 0.2). (*e*) A graph shows the symmetry values for ring closure in 21 cells, ranging from symmetric (less than 0.2, *n* = 1) to asymmetric (between 0.2 and 0.6; *n* = 13/21) and highly asymmetric (greater than 0.6; *n* = 7/21). (*f*,*g*) Graphs show the fluorescence intensity of mNeonGreen-anillin along the ingressing (*f*) and non-ingressing (*g*) sides of the cortex of an asymmetrically dividing MDCK cell starting 2 min before anaphase onset, with timepoints shown in different colours as indicated by the scale.

We also observed that the furrow ingressed asymmetrically in MDCK cells, but not in the other cell lines (figures 4*d* and 6*a*). MDCK cells acquire apicobasal polarity when grown to confluency in culture, however, to facilitate live imaging studies, our cells were not confluent [84]. Since 2D side views of ingression can be misleading, we measured ring closure using end-on-views. To do this, we plotted the position of the ring over time starting before anaphase onset until the ring had closed (e.g. two cells are shown in figure 6*b*,*c*). We also calculated the distance between the centre of the cell at the first timepoint and the centre of the ring at the last timepoint (figure 6*d*). We found that ring closure occurred asymmetrically in 61.9% of cells (symmetry value between 0.2 and 0.6; *n* = 13/21) and highly asymmetrically in 33.3% of cells (symmetry value greater than 0.6; *n* = 7/21). Meanwhile, only one cell (*n* = 1/21) closed symmetrically (symmetry value less than 0.2; figure 6*d*,*e*). We also measured the levels of anillin along the ingressing and non-ingressing cortex in an asymmetrically dividing MDCK cell. We found that anillin was cortical and more broadly distributed along the ingressing versus non-ingressing cortex until about 6 min after anaphase onset, when a peak of similar intensity formed on both sides (figure 6*f*,*g*). The peak on the ingressing cortex was also broader and continued to increase in intensity compared to the non-ingressing cortex (figure 6*f*,*g*). It is exciting to speculate that the asymmetric distribution of anillin could influence ring closure in these cells, and the lack of junctions to transmit forces between cells suggests that anillin distribution and asymmetric ingression are controlled intrinsically.

## 3. Discussion

In this study, we reveal cytokinetic diversity between different cell types by endogenously tagging cytokinesis proteins with fluorescent tags and visualizing them by live imaging. Specifically, we successfully tagged endogenous anillin, Ect2 and RhoA with mNeonGreen and visualized their localization for the first time in live human cells, enabling robust comparative studies of these key cytokinesis regulators. A limitation of gene editing tools is the risk of introducing undesired off-target mutations. To alleviate this issue, we used the high-specificity HypaCas9 variant, which has the advantage of preserving on-target editing while reducing off-target cutting [85,86]. Several approaches have been devised to reduce the frequency of off-target cutting by Cas9 and the frequency of mutagenic repair, including the use of paired Cas9 nickases or Cas9-sgRNA ribonucleoprotein complexes [87,88]. These improvements on CRISPR/Cas9 gene editing tools help generate high-quality engineered cell lines to ensure robust biological results without artefacts.

To date, most cytokinesis proteins have been studied using over-expressed transgenes in HeLa cells, which may influence the interpretation of their function. Additionally, probes to visualize RhoA or its GEF Ect2 have been notoriously difficult to use [9,14,26,61,62]. Comparing the spatio-temporal localization of endogenous RhoA, Ect2 and anillin revealed that their enrichment at the equatorial cortex occurs at similar times and aligns with the timing of ring assembly and ingression. However, while anillin and RhoA broadly localize along the equatorial cortex, Ect2 localizes to a narrower region, suggesting differences in how the cortical localization of Ect2 is controlled compared to anillin and RhoA. Additionally, Ect2 is first visible at the central spindle, which supports prior findings that an Ect2–Cyk4 complex first forms at the central spindle during mitotic exit and is then released or recruited to the overlying membrane. The localization of anillin and Ect2 is largely in agreement with previous studies done in HeLa cells using immunofluorescence with specific antibodies. Given that fixation can cause artefacts and that epitopes may not be consistently accessible, it was not known whether immunofluorescence provided an accurate picture of their localization pattern. Our findings clarify any variability in localization that may have been reported due to differences in chemical fixation and cell cycle stage (e.g. [9,15,63]). Crucially, this is the first study to our knowledge that reveals the localization of pools of endogenous RhoA during cytokinesis, which was predicted to be enriched at the equatorial membrane based on immunofluorescence after TCA fixation [9,14,63]. Interestingly, RhoA was visibly enriched at the equatorial cortex after anillin. While the cytosolic pools of RhoA could obscure weaker cortical signals, this timing could also reflect the positive feedback between the two proteins, with anillin helping to cluster PI_4,5_P_2_ lipids that RhoA preferentially binds to, and by increasing the residence time of RhoA at the cortex [19]. There is also the caveat that N-terminally tagged RhoA may not be fully functional. However, its ability to respond to Ect2 over-expression and to accumulate at the equatorial cortex suggests that it is at least partially functional.

Our knowledge of mammalian cytokinesis is largely derived from studies of HeLa cells, but the mechanisms regulating cytokinesis are expected to vary with fate, size, ploidy and geometry. Here, we reveal cytokinetic diversity by comparing the spatio-temporal localization of endogenously tagged anillin between different cell lines. This includes HeLa cells as well as cell lines where cytokinesis has not been well-studied (HEK293, MDCK, HepG2, HCT116). We observed differences in (i) the duration of ingression, (ii) the timing and breadth of the cortical localization of anillin, (iii) the amount of anillin in the furrow and (iv) how symmetrically the ring closes.

**Figure 7.**
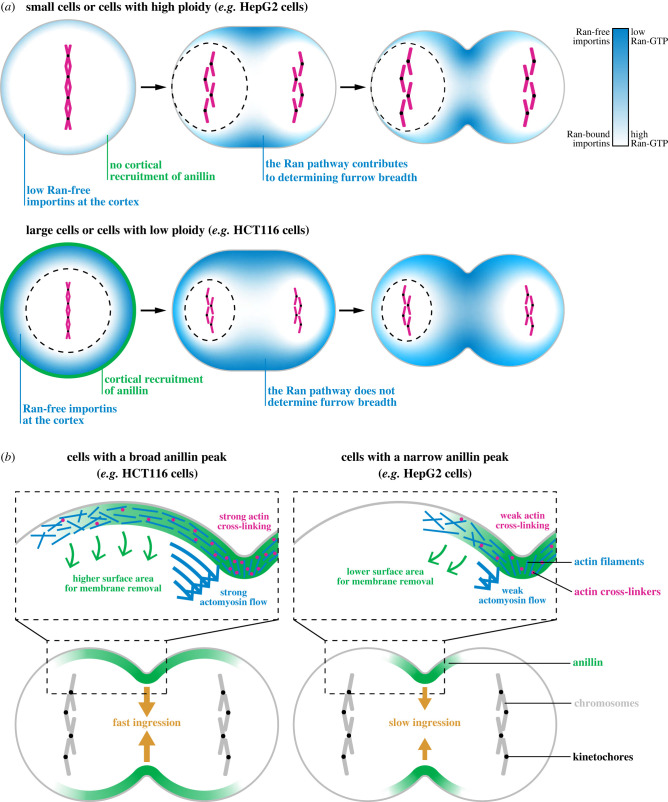
Mechanisms controlling ring assembly and constriction in human cells. (*a*) Cartoon schematics show two different cell types in metaphase, anaphase and telophase, with the position and strength of Ran-GTP (white) and Ran-free importin (blue) levels. In small cells or cells with high ploidy (top), Ran-GTP levels reach the cortex, restricting where Ran-free importins can bind to anillin. In large cells or cells with low ploidy (bottom), Ran-GTP levels are too low to reach the cortex and Ran-free importins can globally bind to and recruit anillin, requiring other pathways to control ring position. (*b*) Cartoon schematics show how the breadth of anillin could influence ring closure. In cells where anillin is more broadly localized (left), there is more surface area for the removal of membrane microdomains by anillin–septin complexes, or there could be stronger actomyosin flows, and/or greater cross-linking of actin filaments to facilitate their alignment for faster ingression compared to cells where anillin is more narrow (right).

The timing of anillin's cortical localization varies among the different cell types. Anillin is cortically localized before anaphase onset in HEK293, HCT116 and MDCK cells, but not in HeLa and HepG2 cells. Previous studies reported that anillin is cortically localized during metaphase in BHK-21 (baby hamster kidney) and *Drosophila* S2 cells, but this was not investigated further [21,89,90]. Based on recent studies showing that importin-binding regulates the cortical localization of anillin, we propose that ploidy relative to cell size or chromatin position could determine whether importins reach sufficient levels to recruit anillin to the cortex in metaphase (figure 7*a*) [38,39,44]. Importins bind to nuclear localization signals (NLS) in proteins, which can be competed off by Ran-GTP [91,92]. The RanGEF RCC1 is bound to histones and RanGAP is in the cytosol, causing Ran-GTP to be enriched around chromatin. The strength of this gradient and whether Ran-GTP levels are high enough to impact importin-binding to cortical NLS-proteins would determine if anillin is localized cortically or not (figure 7*a*) [91,92]. Thus, our model is that in cells that are small and/or have higher ploidy (e.g. HeLa, HepG2), importins cannot recruit anillin to the cortex during metaphase, but can recruit anillin to the equatorial cortex during anaphase as the chromosomes segregate (figure 7*a*, top). Thus, the Ran pathway could be a dominant mechanism that controls the breadth of anillin in these cells. In cells that are large and/or have lower ploidy (e.g. HCT116), Ran-free importins reach sufficient, uniform levels to recruit anillin to the cortex during metaphase (figure 7*a*, bottom), and other mechanisms determine the breadth of anillin during anaphase. Our model also has implications for cells where chromatin is asymmetrically positioned, which could lead to the asymmetric localization of cortical anillin and influence filament organization for ingression (e.g. MDCK cells) [4,5,38,39]. Other mechanisms could also directly or indirectly control the localization of anillin in the different cell lines. Cells with higher ploidy would have more kinetochores and associated Sds22 that could increase PP1 phosphatase activity to downregulate ERM proteins (ezrin, moesin, radixin) and promote the removal of F-actin from the nearby cortex [45]. Astral-dependent machinery could also be expressed at different levels and impact the cortical localization of anillin (e.g. [34,36]). These mechanisms could be tested using the reagents described here to study cytokinesis in combination with perturbations that selectively interrogate these pathways.

We also observed that the breadth of cortical anillin at furrow initiation varies among the different cell lines. Anillin is distributed broadly along the equatorial cortex in HCT116 cells, while it is narrow in HepG2 cells compared to the other cell lines. Interestingly, HCT116 cells also ingress faster and HepG2 cells take longer to ingress than other cell lines, suggesting a correlation between the breadth of anillin localization and the efficiency of ring closure. Importantly, this correlation does not reflect the *amount* of anillin in the furrow, rather how it is distributed. A recent hypothesis paper suggested that an anillin–septin complex controls the removal of membrane microdomains from the ring to relieve tension during closure [20]. The model proposed by the Hickson laboratory would explain our findings as the broader distribution of anillin in HCT116 cells (figure 7*b*, left) could promote more efficient ring closure by providing a greater surface area for this ‘outflow’ and removal of microdomains compared to HepG2 cells (figure 7*b*, left) [20]. However, the breadth of anillin could also reflect differences in other mechanisms that have been experimentally shown to facilitate ingression by promoting the alignment of actin filaments. These mechanisms include cortical flows in the axial and equatorial axis, and the distribution of actin cross-linkers [93–98]. Future studies are required to test these models and to reveal how anillin localization affects ring closure kinetics.

We also found that ring closure occurs asymmetrically in MDCK cells. Although these cells establish apicobasal polarity in culture when grown to confluency [84], we imaged these cells at lower confluency, revealing that intrinsic mechanisms control ring ingression. Few studies have explored the mechanisms controlling the symmetry of ring closure, which is not well understood [44,49,99–101]. The ring may close at least partially asymmetrically in many cell types, and the extent of asymmetry could reflect differences in cell size and fate in addition to adhesion to neighbouring cells [99]. For example, in the two-cell *C. elegans* embryo, ring closure occurs asymmetrically in the larger AB daughter cell fated to become somatic tissue, but not in the smaller P_1_ cell fated to become the germline [44]. Inducing changes in cell size or perturbing the Ran pathway affects the symmetry of ring closure in P_1_ cells, suggesting that multiple factors control this process [44]. The nature of the ring may lend itself to asymmetric closure. As the ring pulls in part of the membrane, curvature and flows are induced that promote actomyosin filament alignment and improve energy-efficiency for faster ingression in that location [100]. However, the asymmetric ingression we observed in MDCK cells was more extreme compared to the other cell lines. A recent study showed that apical PAR proteins localize mutually exclusively to anillin in cells within developing mouse embryos suggesting they could compete for the same filamentous networks [49]. These proteins could be asymmetrically distributed prior to establishing full apicobasal polarity, which could cause anillin to be more broadly distributed along the asymmetrically ingressing cortex (e.g. Figure 6*a*). However, the mechanisms controlling their distribution in cells prior to establishing polarity are not clear, nor is the biological significance of asymmetric closure.

In addition to generating new tools and fundamental knowledge of cytokinesis, our work provides methodologies for more consistent analysis of cytokinesis. Studies of cytokinesis tend to differ in how parameters are measured and reported. Previous studies in *C. elegans* provide a strong foundation for methodologies that could be universally applied to other cell types (e.g. [44,101–103]), including the macros used in this study, which are publicly available. We also expanded on the CRISPR/Cas9 reagents generated by the Allen Institute for Cell Science by tagging multiple cellular components with different fluorescent proteins, which will be useful for studies of cytokinesis and other biological processes in human cells. All the reagents used to generate the endogenously tagged cell lines in this study are available to the community through Addgene. Combining the use of endogenous tags with quantitative measurements will help capture cytokinetic diversity across a broader range of mammalian cell types and generate new knowledge of the mechanistic differences regulating cytokinesis.

## 4. Material and methods

### 4.1. Cell culture

HEK293, HeLa and MDCK cells were cultured in Dulbecco's modified Eagle medium (DMEM; Wisent) media supplemented with 10% Cosmic calf serum (CCS; Cytoviva). HCT116 cells were cultured in McCoy's media (Wisent) supplemented with 10% CCS. HepG2 cells were cultured in Eagle's minimum essential medium (EMEM; Wisent) supplemented with 10% fetal bovine serum (FBS; Cytoviva). The cells were maintained in 10 cm dishes in incubators at 37°C with 5% CO_2_ as per standard protocols [38,39]. For long-term storage, cells were washed and resuspended in freezing media (50% FBS, 40% DMEM or EMEM media and 10% DMSO) and stored in liquid nitrogen.

### 4.2. Cloning

To generate the pX459-HypaCas9-mRuby2 CRISPR construct (Addgene no. 183872), we digested the pX459V2.0-HypaCas9 plasmid (obtained from [86]; Addgene no. 108294) with EcoRI (New England Biolabs) and used the GeneJET gel extraction kit (ThermoFisher Scientific) to extract the backbone as per manufacturer's protocols. To prevent re-ligation the backbone was dephosphorylated with Antarctic phosphatase (New England Biolabs) as per manufacturer's instructions. The mRuby2-T2A-Puro insert was generated by amplifying the mRuby2 and Puromycin resistance genes with shared overlap, and assembling them by SOEing (Splicing by Overlap Extension) PCR. This was done by first performing a Phusion PCR reaction (ThermoFisher Scientific) without primers for 15 cycles, then adding primers to the reaction for amplification during the remaining 25 cycles. The mRuby2-T2A-Puro PCR product was then purified and digested with EcoRI for ligation into the dephosphorylated backbone. Clones were screened by PCR and validated by sequencing the insert region (Eurofins Operon).

The sgRNA spacer sequences were selected using Benchling [104,105] and CCTop [106] for the ANLN, ECT2, RHOA and MYH10 genes and the AAVS1 locus. Multiple spacers were selected for each target site. One of the AAVS1 spacer sequences was obtained from Oceguera-Yanez *et al*. [107] and the spacer sequences for the H2BC11, ACTB, MYH10 and TUBA1B genes were obtained from the Allen Institute for Cell Science [76] (Allencell.org). All sgRNA spacers tested are listed in electronic supplementary material, table S1. The sgRNA spacers were cloned into the pX459V2.0-HypaCas9 and the pX459V2.0-HypaCas9-mRuby2 plasmids using previously described methods [108]. Briefly, two complementary oligos containing the spacer sequence were designed as follows (where (N)_20_ corresponds to the 20-nucleotide spacer sequence or its reverse complement):

Forward oligo: 5′-CACCG(N)_20_-3′
Reverse oligo: 5′-AAAC(N)_20_C-3′

Oligos were annealed and ligated into the backbone (pX459V2.0-HypaCas9 or pX459V2.0-HypaCas9-mRuby2) pre-digested with BbsI (New England Biolabs). Two clones were selected for each sgRNA and sequenced to verify the spacer sequence.

To build the repair templates, the homology arms were first amplified from HEK293 genomic DNA extracted using the Qiagen DNeasy Blood and tissue kit or plasmid DNA by PCR. For genome sequences that were recalcitrant to PCR amplification, we used touchdown PCR conditions [109] with 1X GC buffer or 0.5X HF buffer instead of 1X HF buffer with Phusion polymerase (ThermoFisher Scientific). The fluorescent tags (mNeonGreen, mRuby2 and TagBFP) were amplified independently using primers that were designed to introduce overlap with the homology arms (15–51 bp of overlap). The primers used to clone the repair templates are listed in electronic supplementary material, table S2. The repair templates were assembled by SOEing PCR as described above, then blunt-end ligated into the pJET1.2 vector using the CloneJET PCR cloning kit (ThermoFisher Scientific). Multiple clones were screened by PCR and sequencing.

For transgene expression, the coding sequences for anillin, Ect2 and Ect2(C-term) were cloned into the Golden Gate entry vector pYTK001 (obtained from [110]; Addgene no. 65108). They were then fused to mNeonGreen (Allele Biotech) or mScarlet-I (obtained from [111]; Addgene no. 98839) and assembled into a custom Golden Gate expression vector (pGG). The Golden Gate assembly reaction was carried out using BsaI (New England Biolabs) following standard protocols [110]. Isolated colonies were picked and screened by PCR and sequencing.

### 4.3. Transfection, nucleofection and NHEJ inhibition

To improve editing efficiency, all cells were treated with NHEJ inhibitors NU7441 (2 µM; Tocris Bioscience) and SCR7 (1 µM; Xcess Biosciences) 4 h before transfection and for 48 h after transfection. HEK293, HeLa and HepG2 cells were seeded in 24-well dishes to reach 60% confluency on the day of transfection. Cells were transfected using Lipofectamine 3000 and P3000 reagent (ThermoFisher Scientific) according to manufacturer's instructions. Plasmids were introduced into HCT116 and MDCK cells using nucleofection with the 384-well HT Nucleofector and the SE cell line kit (Lonza) as per manufacturer's instructions. The cells were nucleofected using the EN-113 program for HCT116 cells, and the CA-152 program for MDCK cells, and the amount of repair template and plasmid were adjusted per cell type.

For transient transgene expression, vectors (pGG-mNG-Anillin, pGG-mNG-Ect2, pGG-mScar-Ect2(C-term) and GFP-RhoA) were transfected into HeLa cells plated on coverslips and grown to 60% confluency, using Lipofectamine 3000 and P3000 reagent (ThermoFisher Scientific) as per manufacturer's instructions.

### 4.4. Fluorescence-activated cell sorting

Tagged cells were isolated or enriched by FACS 7–14 days after transfection. The cells were resuspended thoroughly in FACS buffer comprised 1 mM EDTA, 25 mM HEPES pH 7.0 and 1% FBS in PBS buffer, then passed through a 35 µm strainer to remove large cell clumps and transferred to 5 ml FACS tubes for sorting. Cells were sorted using a FACSMelody cell sorter (BD Biosciences) with gates set to capture individual fluorescent cells. The fluorescent cells were sorted into individual wells of a 96-well plate containing recovery media (media supplemented with 20% FBS and 1X penicillin-streptomycin; 50 units ml^−1^ penicillin and 50 µg ml^−1^ streptomycin; Wisent). Alternatively, fluorescent cells were enriched by sorting 15 000 cells into a FACS tube with recovery media. The enriched population was then resuspended in fresh recovery media and seeded into one well of a 96-well plate. The cells were left to recover, and media was supplemented as needed. For flow cytometry, cells were prepared using the same protocol and measured on different days after transfection. Flow cytometry data were acquired using the FACSMelody cell sorter and analysed with the R package CytoExploreR [112].

### 4.5. Genotyping

For genotyping, we first determined whether the tag was inserted into the target locus using PCR. Clones were expanded by splitting cells from individual colonies in 96-well plates into 48-well plates, and subsequently into 24-well plates. The target locus was amplified directly from the cells using the Phire Plant Direct PCR mastermix (ThermoFisher Scientific) in three junction PCR reactions as per manufacturer's protocols. The primers used for these reactions are listed in electronic supplementary material, table S3. The three PCR reactions were designed to amplify the wild-type (WT) locus as well as the left and right junctions of the integration sites. Touchdown PCR was used to improve the quality of the PCR products. Six to twelve positive clones were then expanded into 10 cm dishes for further genotyping. Genomic DNA was extracted using the Qiagen DNeasy Blood and tissue kit, then used to amplify the target locus with Phusion polymerase (ThermoFisher Scientific) by touchdown PCR. The genotype was verified by extracting PCR products and sequencing the tagged and untagged alleles. When possible, homozygous clones carrying only tagged alleles were selected. Alternatively, heterozygous clones carrying a tagged allele and a wild-type allele were selected.

### 4.6. Microscopy

The endogenous tags and ectopic fluorescent protein expression were imaged using microscopy. Cells grown on 6-well plates were imaged 2 and 10 days after transfection on a Leica DMI6000B inverted epifluorescence microscope with filters for the appropriate wavelengths, using a 20×/0.35 NA objective, an Orca R2 CCD camera (Hamamatsu) and Volocity software (PerkinElmer). Single-cell-derived clones were initially screened for fluorescence and localization 7–10 days after FACS isolation using a Nikon Eclipse TS100 microscope equipped with a DS-Qi1Mc camera the 10×/0.25NA objective. Clones with uniform expression and expected cellular localization were selected for further screening.

To image cells during cytokinesis, tagged HeLa, HCT116, HepG2 and MDCK cell lines were seeded onto acid-etched round coverslips (25 mm, no. 1.5) in 6-well plates and grown to 70% confluency. The coverslips were transferred to a magnetic chamber (Quorum) with 1 ml media before imaging. Tagged HEK293 cells were seeded directly onto 4-well μ-slides (Ibidi) for imaging. To visualize chromatin, Hoechst 33342 (Invitrogen) was added to the cells at a final concentration of 0.4 µM for 30 min prior to imaging. Imaging was performed using an inverted Nikon Eclipse Ti microscope (Nikon) equipped with a Livescan Sweptfield scanner (Nikon), Piezo Z stage (Prior), IXON 879 EMCCD camera (Andor), and 405, 488 and 561 nm lasers (100 mW, Agilent) using the 100×/1.45 NA objective. The cells were kept at 37°C and 5% CO_2_ during imaging in an INU-TiZ-F1 chamber (MadCityLabs). Z-stacks of 1 µm thickness were acquired every 1–2 min using NIS Elements software (Nikon, v.4.0).

To collect images of the over-expressed transgenes, the coverslips were transferred to a magnetic chamber with 1 ml of media 1 day after transfection, and imaged on a Leica DMI6000B inverted epifluorescence microscope with filters for the appropriate wavelengths, a 10×/0.25 NA objective, an Orca R2 CCD camera (Hamamatsu) and Volocity software (PerkinElmer), or a Nikon Eclipse TiE inverted epifluorescence microscope using LEDs in the appropriate wavelength with an Evolve 512 EMCCD camera (Photometrics) and NIS Elements acquisition software (Nikon) using the 60×/1.4 NA objective.

### 4.7. Cell viability assay

To monitor the growth of the different cell lines, we used the WST-8 cell proliferation assay kit (Cayman Chemical), as per manufacturer's instructions. For each cell line, 10 000 cells were seeded per well in a 96-well plate and viability was assayed on days 0, 1, 2, 3 and 5, for a total of approximately 4 population doubling times. For the assay, the electron mediator solution and the WST-8 developer reagent were mixed in equal parts, then added to each well and incubated at 37°C for 2 h. After this, the absorbance at 450 nm was measured on a TECAN Infinite M200 plate reader. The assay was repeated in triplicate for each cell line.

### 4.8. Image analysis

Linescans were performed and measured for each tagged cell line using Fiji. All images acquired using NIS Elements (Nikon) were opened in Fiji (v.2.3, NIH) and analysed using a macro modified from Ozugergin *et al*. [44]. Briefly, the macro was designed to isolate the green channel from the movie file, subtract background signal and perform a bleach correction. The desired timepoint and Z slices were entered manually, and the macro generated a Z-stack average projection. A five-pixel-wide line was then traced along the cortex of the cell, from one pole to the other, along with a straight one-pixel-wide line intersecting the furrow. The macro then measured the fluorescence intensity of each pixel along the length of the linescan, and positioned the pixels in relation to the furrow. The average intensity projection of two central Z slices was used for cortical linescans, while six central slices were used to measure the central spindle in Ect2-tagged cells. All data were exported for use in Excel (Microsoft) and Prism (v.9.3, GraphPad) for further analysis. Pixel intensity values were normalized by subtracting the average baseline intensity (calculated as the average intensity of the first or last 50 pixels of the linescan) from the maximum intensity value. To obtain breadth measurements, the number of pixels above 50% or 75% of the peak value were counted and converted to microns. Intensities above 50% were used to compare the breadth of RhoA to Ect2 and anillin (figure 3; electronic supplementary material, figure S4A) to accommodate the relatively low peak intensity levels for RhoA, while intensities above 75% were used to compare anillin across cell lines (figure 4; electronic supplementary material, figures S4B and S5). Pixels outside of the peak region that had intensities higher than the cutoff value were excluded from these calculations. Enrichment at the equatorial (furrow) versus polar cortex was calculated as a ratio between the average pixel intensity in the breadth and the poles (the first or last 50 pixels in the linescans). To measure the ratio of the cortical to cytosolic anillin in metaphase cells, the average intensity of the signal at the cortex was measured by a linescan drawn around the cortex using the macro described above, while the average intensity of the cytosol was measured by drawing a region of interest.

Ring closure was measured as described in Ozugergin *et al*. [44]. In short, a 250 × 50-pixel area containing the ring was rotated using SciKit Image (v.0.16.2) to generate an end-on view of the ring. The outline of the ring was drawn manually as an ellipse in FIJI and the coordinates were noted. Best-fit circles for each ellipse were plotted using Python 3 and the Jet colormap. The centre of the ring in the first timepoint was set to 0,0 and the radius was set to 1, and ellipse coordinates in the subsequent timepoints were normalized to the first timepoint. Symmetry values were obtained using the Pythagorean theorem to calculate the distance between the centre of the cell in the first timepoint and the centre of the ring in the last measured timepoint. Cells with values less than 0.2 (close to 0) were defined as having symmetric ring closure, while cells greater than 0.2 and greater than 0.6 were considered to have asymmetric and very asymmetric ring closure, respectively.

### 4.9. Statistical analysis

Box and whiskers plots were generated using Prism (v.9.3, GraphPad) to show median values (central line), quartiles (box edges) and minimum and maximum values (whiskers). Statistical significance was tested using a Brown–Forsythe and Welch's ANOVA, followed by multiple comparisons using Dunnett's T3 test, or by Welch *t*-test (GraphPad Prism v.9.3). Significance levels were defined as: *p* > 0.5 non-significant (n.s.), * *p* ≤ 0.05; ** *p* ≤ 0.01; *** *p* ≤ 0.001; ^#^ *p* ≤ 0.0001.

## Data Availability

All plasmid sequences are available on Addgene. The data are provided in the electronic supplementary material [113].
